# Investigation of Antibacterial and Fouling Resistance of Silver and Multi-Walled Carbon Nanotubes Doped Poly(Vinylidene Fluoride-co-Hexafluoropropylene) Composite Membrane

**DOI:** 10.3390/membranes7030035

**Published:** 2017-07-13

**Authors:** Lutendo E. Macevele, Kgabo L. M. Moganedi, Takalani Magadzu

**Affiliations:** 1 Department of Chemistry, University of Limpopo, Private Bag x1106, Sovenga 0727, South Africa; Lutendo.Macevele@ul.ac.za; 2 Department of Biochemistry, Microbiology and Biotechnology, University of Limpopo, Private Bag x1106, Sovenga 0727, South Africa; Kgabo.Moganedi@ul.ac.za

**Keywords:** composite membranes, multi-walled carbon nanotubes, PVDF-HFP, fouling resistance, antibacterial activity

## Abstract

Composite membranes were successfully prepared using a phase-inversion method. The X-ray powder diffraction (XRD) and energy dispersive X-ray (EDX) profiles has confirmed formation of 4.8 wt % Ag/poly(vinylidene fluoride-co-hexafluoropropene) (PVDF-HFP), 3 wt % Ag-MWCNTs/PVDF-HFP (EDX surface composition of Ag nanoparticles) and 1.5 wt % MWCNTs/PVDF-HFP composite membranes. The MWCNTs crystallites are mainly encapsulated by a layer of PVDF-HFP, as evidenced by disappearance of graphitic peak. The scanning electron microscopy (SEM) images have depicted the formation of microporous structure, with few MWCNTs on the surface and strongly interacting with PVDF-HFP as demonstrated by thermogravimetric analysis (TGA), XRD and Fourier transform infrared (FTIR) data. The data indicated an increase in porosity, swellability and water content of the PVDF-HFP membrane with the addition of MWCNTs and/or Ag nanoparticles, showing an improved hydrophilicity. The 1.5 wt % MWCNTs/PVDF-HFP composite membrane showed good desalination and fouling resistance rates, which correlates with a low water contact angle. The combined effects of Ag nanoparticles and MWCNTs do not promote fouling resistance of PVDF-HFP membranes, as shown during NaCl microfiltration (this is linked with high water contact angle as compared to that of MWCNTs/PVDF-HFP composite). Both 1.5 wt % MWCNTs/PVDF-HFP and 3 wt % Ag-MWCNTs/PVDF-HFP composite membranes prevented the bacteria passing through the membrane (100% bacterial load reduction). The surface of 3 wt % Ag-MWCNTs/PVDF-HFP showed good bactericidal and non-leaching properties of the dopant materials (MWCNTs and Ag), as evidenced by bacterial growth on the edges of the membranes.

## 1. Introduction

Clean drinking water availability is a major problem for developing countries [1]. Scarcity of safe drinking water remains a global problem and is expected to rise with increasing population growth and environmental changes [2]. Drinking polluted or contaminated water can cause serious health problems; for example, cholera and diarrheal diseases cause many deaths of children in developing countries [3,4]. The World Health Organization (WHO) recommended that any water intended for drinking should contain fecal and total coliform counts of 0.00 in any 100 mL sample [5]. When bacteria are encountered in water samples, immediate investigative action should be taken [5].

Membranes technology has become a popular filtration technique worldwide and is very important in removing organic and inorganic pollutants [6]. An example of one commonly used membrane is poly(vinylidene fluoride) (PVDF). PVDF is a semi crystalline thermoplastic material possessing good chemical resistance, high hydrophobicity, good mechanical strength and outstanding resistance to corrosion from many chemicals and organic solvents, etc. [7,8]. Although many reports exist on the developments of PVDF membranes for wastewater treatment, their fabrication remains a challenge [9]. This is mainly due to their low interfacial stability [10].

Among various membrane compositions, none of the studies consider doping nanomaterials on poly(vinylidene fluoride-co-hexafluoropropene) (PVDF-HFP) for water treatment purposes [9,11]. This polymeric membrane possesses a high dielectric constant and good mechanical properties [10]. It is a chemically inert fluoropolymer, with lower crystallinity compared with PVDF [12]. This is due to the combination of an amorphous phase of hexafluropropylene (HFP) into the vinylidene fluoride (VDF) blocks, which aids in higher ionic conduction of the polymer, whereas the crystalline phase acts as a mechanical support for the polymer [13]. Studies have shown that addition of MWCNTs improves the electrical, mechanical and thermal properties of polymers [14,15]. This carbon nano-material was further reported to inactivate bacteria upon direct contact [16,17]. Silver (Ag) nanoparticles have been extensively researched, due to their high antibacterial activity [18,19,20] etc. For example, Ag loaded membranes displayed an improved surface hydrophilicity [21] and good antibacterial activity [11,22]. However, the latter were releasing Ag nanoparticles as evidenced by the formation of a clear halo zone around the membranes.

Herein, the study has developed and investigates the properties of composite membranes based on PVDF-HFP polymer modified with either silver nanoparticles and/or MWCNTs. The investigation focused on the role of MWCNTs and Ag nanoparticles on fouling resistance, desalination and antibacterial activity of the composite membrane.

## 2. Material and Method

### 2.1. Materials

PVDF-HFP, *N*,*N*-dimethylacetamide (DMAc, analytical grade), Silver nitrate (AgNO_3_), Nitric acid, Sulphuric acid, MWCNTs, Polyethylene glycol (PEG), Polyvinylpyrrolidone (PVP), and Sodium dodecyl sulphate (SDS) were all purchased from Sigma Aldrich, Darmstadt, Germany.

### 2.2. Preparation of Ag/MWCNTs

Acid treated MWCNTs and Ag/MWCNTs were prepared following a method described elsewhere [18]. Briefly, approximately 1 g of MWCNTs was sonicated at 25 °C in a mixture of HNO_3_ and H_2_SO_4_, 1:3 (*v*/*v*). After 3 h of sonication, the acid-treated MWCNTs were diluted with 200 mL of distilled water and filtered through a 0.45 µm pore sized nylon membrane. The acid treated MWCNTs were then washed thoroughly with distilled water until a neutral pH is reached, and dried at room temperature overnight.

For the synthesis of silver nanoparticles, silver nitrate solution (50 mL, 0.1 M) and sodium dodecyl sulphate (SDS) (0.1 g) were used as a metal salt precursor and a stabilizing agent, respectively [23]. Then, 1:1 solutions of hydrazine hydrate (25 mL, 0.1 M) and sodium citrate (25 mL, 0.1 M) were added drop-wise to a mixture of SDS and AgNO_3_ for 2 h while stirring at room temperature. The mixture was left to stir for an additional 48 h, after which the precipitates were filtered and washed with distilled water, acetone and ethanol.

To prepare Ag/MWCNTs, the same procedure described above was followed wherein AgNO_3_ solution (50 mL, 0.01 M) and SDS (0.1 g) was added to a round bottom flask containing 0.2 g MWCNTs.

### 2.3. Preparation of MWCNTs/PVDF-HFP Composite Membranes

Approximately 2.0 g of PVDF-HFP was dissolved in 15 mL of DMAc at 80 °C to form a polymer solution. To this solution, about 0.1 g of PVP and 1 mL of PEG were added (to enhance pore formation) and the reaction mixture was stirred for 2 h at 80 °C [9,24]. Separately, 30 mg of functionalised MWCNTs (f-MWCNTs) was sonicated in 5 mL of DMAc for 30 min.

The final mixture was prepared by adding acid treated MWCNTs to a solution of PVDF-HFP. The mixture was allowed to stir for an additional 1 h and then hand cast into a glass plate using a casting knife (Elcometer 3580 adjustable bird film applicator, BAMR, Cape Town, South Africa) of 180 µm thickness. The prepared membranes were first dried in a vacuum oven at 80 °C (for 30 s) for solvent pre-evaporation and then coagulated using distilled water (at 5 °C) as the anti-solvent. After complete coagulation, the membranes were dried on plain sheets of paper at room temperature.

A similar procedure was followed to prepare membranes consisting of Ag/PVDF-HFP, Ag-MWCNTs/PVDF-HFP composites. For preparation of Ag/PVDF-HFP, sonicated Ag nanoparticles (0.1 g in 5 mL DMAC) were added in the PVDF-HFP solution and for Ag-MWCNTs/PVDF-HFP preparation, sonicated Ag-MWCNTs nanoparticles (0.08 g in 5 mL DMAC) were added to a solution of PVDF-HFP. 

### 2.4. Filtration Studies

One hundred millilitres of sterilised deionised water were spiked with an overnight culture of *E. coli* bacteria, followed by vacuum-filtration through the prepared membranes. The filter membranes were then placed on Nutrient agar plates and incubated for 24 h at 37 °C. Following incubation, the plates were observed for growth on and around the filter membranes for antibacterial and leaching properties. Antibacterial activity was further confirmed by placing a piece of the used membrane in Nutrient broth and observed for growth following an overnight incubation at 37 °C. All experimental procedures were performed in triplicates.

Bacterial enumeration was performed before and after filtration to evaluate the entrapment ability of the membranes. One hundred-fold serial dilutions of the spiked water samples (100 µL) were spread plated on Nutrient agar before filtration treatment, followed by plating 100 µL of the filtrate. Bacterial counts were expressed as colony forming units per millilitres (CFU/mL).

### 2.5. Permeation Tests

#### 2.5.1. Swellability Tests

The membranes were first weighed and then soaked in distilled water for 7 h after which they were weighed again in the balance [25]. The percentage swellability was calculated according to Equation (1):

(1) $$Q_{t}(\%)=\frac{\;(m_{w}/M_{r})}{\;m_{c}}\;\times 100$$

where *m_c_* is the initial mass of the membrane in g, *m_w_* is mass of water absorbed and *M_r_* is the molar mass of water.

#### 2.5.2. Water Content and Porosity Measurements

The membranes where immersed in distilled water for 24 h after which the weight of the wet membrane (*W*_0_) was obtained. The wet membrane was then dried in an oven at 80 °C for 24 h after which it was weighed (*W*_1_) to obtain the dry weight [26]. The water content was obtained using Equation (2):

(2) $$\mathrm{water}\;\mathrm{content}\;(\%)=\frac{\;(W_{0}-W_{1})}{\;W_{0}}\;\times^{}100$$

The porosity was calculated according to Equation (3):

(3) $$P\;(\%)=\frac{\;(W_{0}-W_{1})}{\;Adh}\;\times 100$$

where *A* is the membrane surface area (cm^2^), *d* is the density of water at 25 °C and *h* is the membrane thickness (mm) [27].

#### 2.5.3. Contact Angle (Sessile-Drop Method)

Hydrophilicity of the membranes was quantified by measuring the contact angle that was formed between the membrane surface and water. Contact angles were determined with a Data Physics Optical contact angle analyser (OCA 15EC, Data Physics, Filderstadt, Germany). All contact angle measurements were made using 2 µL of deionised water. To minimise the experimental error, droplets were contacted with the membrane at five random locations for each sample and the average was reported. All measurements were carried out at 25 °C.

### 2.6. Salt Rejection Tests

Desalination tests using composite membranes were carried out for 120 min. All the membranes had an effective area of 0.00126 m^2^. The pervaporation desalination performance of PVDF-HFP membranes was evaluated by measuring water flux and salt rejection. The water flux (*J*) was determined from permeate volume (*V*) measured in (*l*), the effective membrane area (*A*) and the time (*t*) necessary for the volume to be collected [28]. It was measured using Equation (4):

(4) $$J=\frac{\;V}{\;At}\;$$

The membrane salt rejection was then determined based on Equation (5) as reported in literature [29].

(5) $$Salt\;rejection=1-\frac{\;(Conductivity_{permeate})}{\;(\;Conductivity_{feed})}\;\times 100$$

### 2.7. Membrane Characterisation

Thermogravimetric analysis (TGA) was used to measure the change in mass of membrane samples over a range of temperatures. Fourier transform infrared spectroscopy (FTIR) was used to investigate the functional groups present in the membranes. Scanning electron microscopy (SEM) was used to investigate the morphology of the membrane and the energy dispersive X-ray (EDX) was used for elemental analysis. X-ray powder diffraction (XRD) was used to examine the crystallinity of the Ag/MWCNTs. Inductively coupled plasma optical emission spectrometry (ICP-OES) was used to determine trace amounts of silver in the filtered water.

## 3. Results and Discussion

### 3.1. Thermal and Structural Properties of Composite Membranes

**Figure 1:**
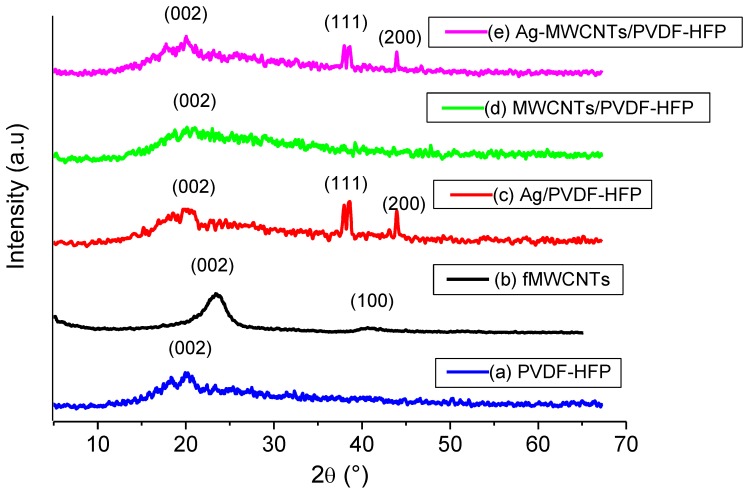
X-ray diffraction patterns of (**a**) poly(vinylidene fluoride-co-hexafluoropropene) (PVDF-HFP), (**b**) fMWCNTs, (**c**) Ag/PVDF-HFP, (**d**) MWCNTs/PVDF-HFP, (**e**) Ag-MWCNTs/PVDF-HFP.

**Figure 2:**
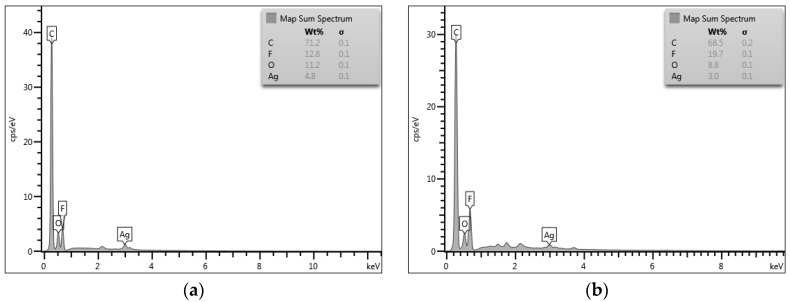
Energy dispersive X-ray (EDX) data of (**a**) Ag/PVDF-HFP and (**b**) Ag-MWCNTs/PVDF-HFP.

Figure 1 shows the XRD patterns of PVDF-HFP, fMWCNTs, Ag/PVDF-HFP, MWCNTs/PVDF-HFP and Ag-MWCNTs/PVDF-HFP membranes. The XRD profile of as-prepared PVDF-HFP shows a noisy hump appearing at 2θ = 20°. The broad peak indexed to (002) crystal plane is ascribed to a graphitic structure of acid treated/functionalised MWCNTs [30]. This broad peak disappears upon addition of 1.5 wt % MWCNTs onto the structure of PVDF-HFP. It is believed that most of the MWCNTs are encapsulated by a layer of PVDF-HFP, hence the disappearance of a peak. The two sharp peaks seen in the XRD profiles of Ag/PVDF-HFP and Ag-MWCNTs/PVDF-HFP membranes are indexed to (111) and (200) planes, confirming the presence of Ag nanoparticles. This was further confirmed by EDX data (Figure 2), which indicated the presence of 4.8 wt % Ag on the surface of PVDF-HFP and 3.0 wt % Ag on the surface of MWCNTs/PVDF-HFP membrane. EDX data further confirmed the presence of fluorine from PVDF-HFP, carbon from both PVDF-HFP and MWCNTs, with oxygen introduced after functionalization of MWCNTs by nitric and sulphuric acid.

**Figure 3:**
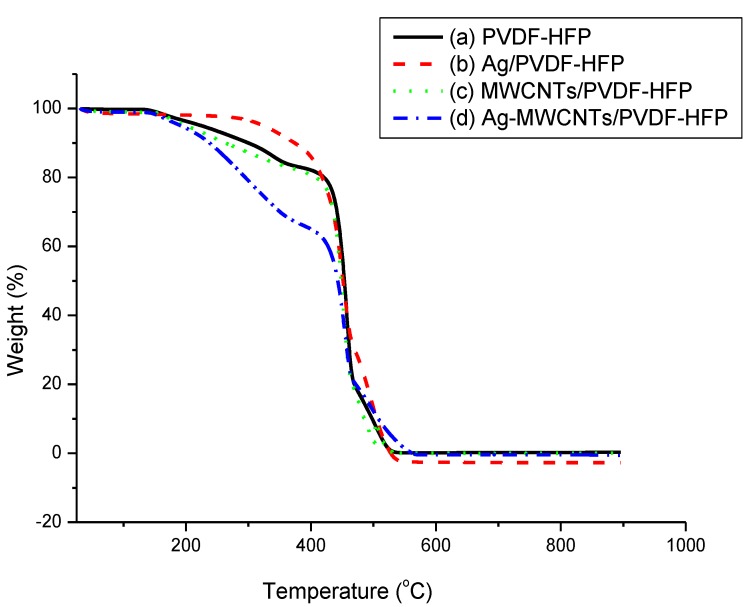
Thermogravimetric analysis (TGA) of (**a**) PVDF-HFP, (**b**) Ag/PVDF-HFP, (**c**) MWCNTs/PVDF-HFP (**d**) Ag-MWCNTs/PVDF-HFP.

Figure 3 shows the thermogravimetric analysis (TGA) of PVDF-HFP, Ag/PVDF-HFP, MWCNTs/PVDF-HFP and Ag-MWCNTs/PVDF-HFP. All membranes remain stable up to 170 °C, with Ag doped PVDF-HFP membrane maintaining stability up to 300 °C, especially when compared to PVDF-HFP polymeric membrane. Both PVDF-HFP and MWCNTs doped PVDF-HFP showed a weight loss of 20% from 170 to 440 °C. The data indicates that MWCNTs do not improve the structural stability of the PVD-HFP membrane, and equally so the structure remains stable in relation to pure PVDF-HFP. Interestingly, the structure of the composites consisting of both Ag nanoparticles and MWCNTs on PVDF-HFP collapsed with a weight loss of 40% from 170 to 450 °C. However, all composites have shown stability within the limits of the daily temperature of water and can easily withstand purification of boiled water. Similar behaviour in terms of structural instability of polymeric membranes (in the presence of dopants) is comparable to the work reported in the literature [31,32].

**Figure 4:**
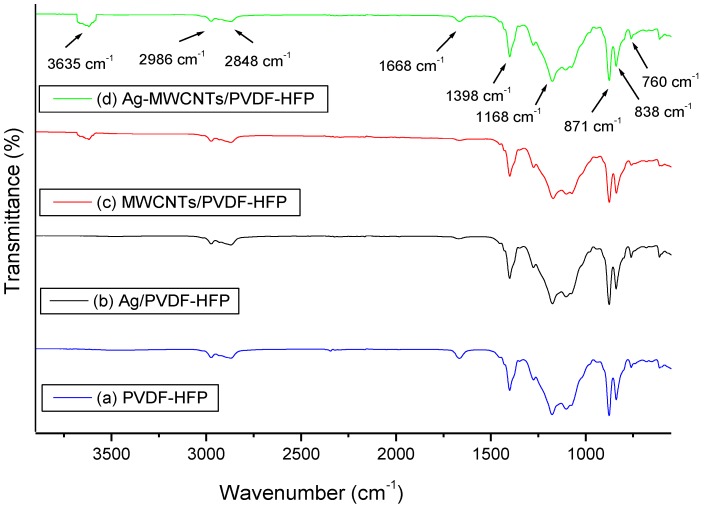
Fourier transform infrared (FTIR) spectra of (**a**) PVDF-HFP, (**b**) Ag/PVDF-HFP, (**c**) MWCNTs/PVDF-HFP, and (**d**) Ag-MWCNTs/PVDF-HFP.

Figure 4 shows the FTIR spectra of PVDF-HFP, Ag/PVDF-HFP, MWCNTs/PVDF-HFP and Ag-MWCNTs/PVDF-HFP composite membranes. All composites have shown the presence of γ phase crystalline structure of PVDF-HFP, due to the presence of the absorption species at 760, 838, 871 and 1168 cm^−1^. A similar structure was observed elsewhere, on *N*,*N*-dimethylacetamide casted PVDF-HFP membrane [12]. The FTIR spectra at 3635 cm^−1^ appearing on both MWCNTs/PVDF-HFP and Ag-MWCNTs/PVDF-HFP composites is due to an O–H stretching mode of MWCNTs.

### 3.2. Morphology of PVDF-HFP Composite Membranes Doped with MWCNTs and Ag Nanoparticles

**Figure 5:**
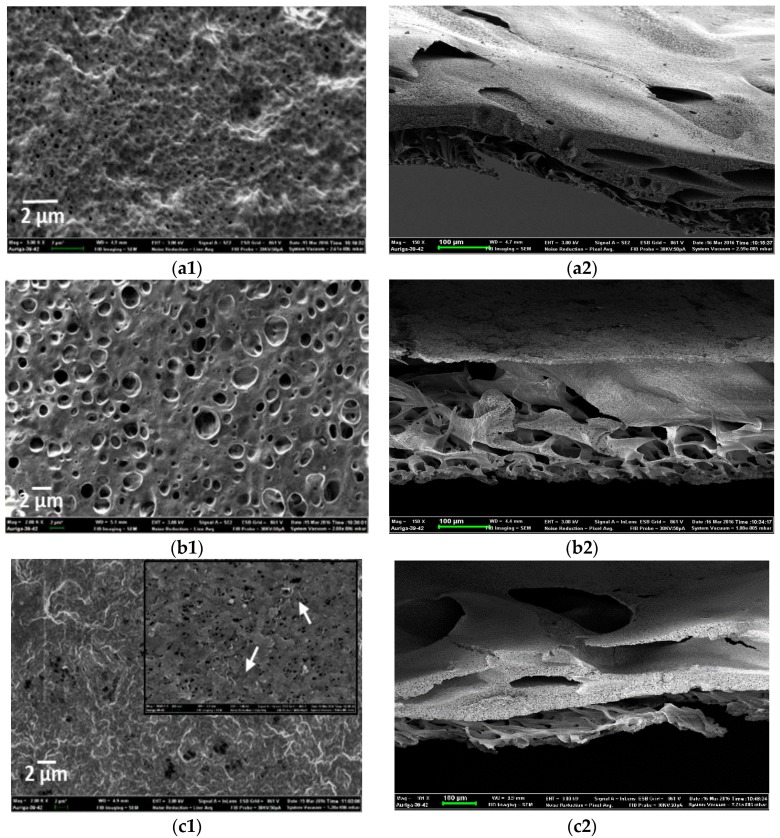

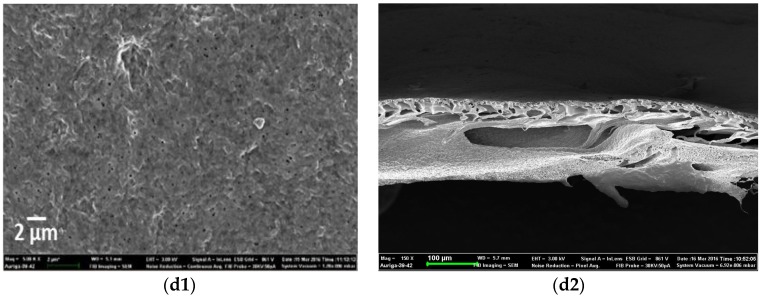
Scanning electron microscopy (SEM) images for top surfaces of (**a1**) PVDF-HFP, (**b1**) Ag/PVDF-HFP, (**c1**) MWCNTs/PVDF-HFP, and (**d1**) Ag-MWCNTs/PVDF-HFP and their cross-sections (**a2**,**b2**,**c2**,**d2**).

Figure 5 shows SEM images of PVDF-HFP, Ag/PVDF-HFP, MWCNTs/PVDF-HFP and Ag-MWCNTs/PVDF-HFP membranes. The addition of Ag nanoparticles changed the spongy surface layer of PVDF-HFP (Figure 5(a1)) into a highly porous structure (Figure 5(b1)). The cross-section clearly depicts a disappearance of a dense layer of PVDF-HFP upon Ag loading (Figure 5(b2)). This data is consistent with the improved permeability of the Ag/PVDF-HFP composite membrane (Table 1), and studies elsewhere on Ag/PVDF membranes [21]. The SEM image in Figure 5(c1), insert, depicts the surface of PVDF-HFP entangled with well dispersed MWCNTs. These MWCNTs can be easily seen on the cross-section (Figure 5(c2)), stretching from the surface to the bottom layer of PVDF-HFP.

The enhanced porosity can be attributed to several openings available on MWCNTs; hence, the MWCNTs/PVDF-HFP composite membrane was found to be highly porous compared to all membranes (Table 1). However, the combined effects of Ag nanoparticles and MWCNTs do not improve the porosity of PVDF-HFP, as evidenced by the formation of a dense surface layer (Figure 5(d1)). Although the cross-section of the MWCNTs/PVDF-HFP (Figure 5(c2)) and Ag-MWCNTs/PVDF-HFP (Figure 5(d2)) membrane are related, the latter had openings and the surface of MWCNTs was blocked by Ag nanoparticles.

#### Physical Properties of Composite Membranes

Table 1 shows the effects of Ag nanoparticles and MWCNTs on swellability, and the water content of PVDF-HFP membrane. The data indicates an increase in swellability and water content of the composite membranes with addition of MWCNTs and/or Ag nanoparticles, showing an improved hydrophilicity. Similar behaviour was reported elsewhere, with Ag nanoparticles improving the hydrophilicity of PVDF membrane [17]. It is worth noting that MWCNTs doped PVDF-HFP had higher porosity as compared to a PVDF-HFP composite containing both Ag nanoparticles and MWCNTs. This is thought to be due to the presence of reactive functional groups on the surface of MWCNTs [33], with the latter possessing smaller pores due to occupation by Ag nanoparticles. Hydrophilicity of the membranes was further confirmed by contact angle measurements.

**Figure 6:**
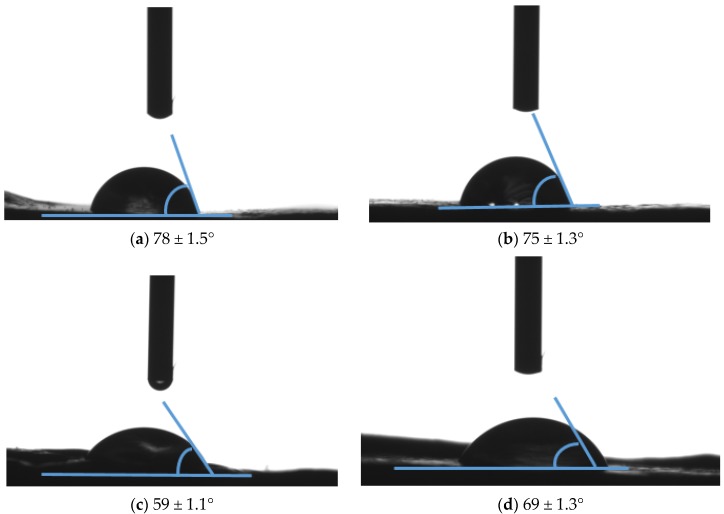
Contact angle measurements of (**a**) PVDF-HFP; (**b**) Ag/PVDF-HFP, (**c**) MWCNTs/PVDF-HFP and (**d**) Ag-MWCNTs/PVDF-HFP.

Figure 6 shows the contact angles of PVDF-HFP, Ag/PVDF-HFP, MWCNTs/PVDF-HFP and Ag-MWCNTs/PVDF-HFP membranes. PVDF-HFP membrane had the highest water contact angle of 78 ± 1.5°, followed by Ag-MWCNTs with 75 ± 1.3°, which is followed by Ag-MWCNTs/PVDF-HFP and MWCNTs/PVDF-HFP with contact angles of 69 ± 1.3° and 59 ± 1.1°, respectively. The data indicates that the hydrophobicity of PVDF-HFP polymeric membrane was reduced when Ag nanoparticles and MWCNTs were added separately. Similar contact angle on 0.4 wt % MWCNTs on PVDF-HFP was reported in the literature [34]. Surprisingly, the combined Ag and MWCNTs nanoparticles could not further lower the hydrophobicity of PVDF-HFP.

### 3.3. Water Filtration Studies

#### 3.3.1. Pure Water Flux Measurement and Antifouling Performance

**Figure 7:**
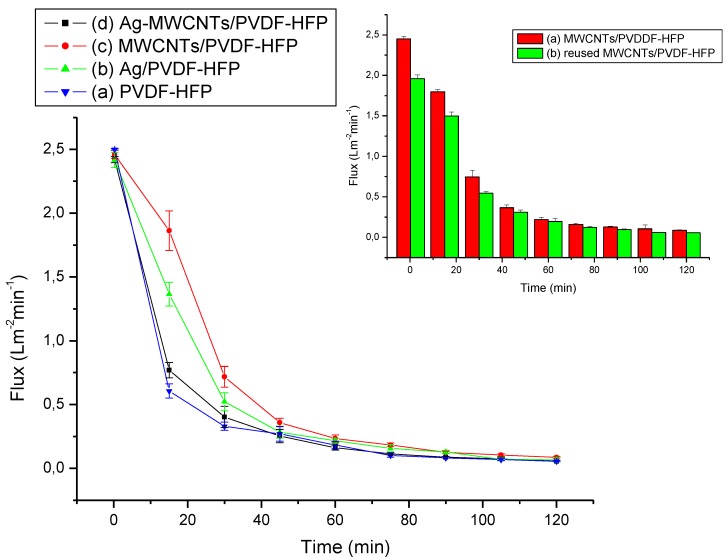
Filtrate flux for salted water (2000 ppm) filtered through the (**a**) PVDF-HFP, (**b**) Ag/PVDF-HFP, (**c**) MWCNTs/PVDF-HFP, and (**d**) Ag-MWCNTs/PVDF-HFP membranes, and the insert showing the 1st and 3rd filtration cycles of filtrate flux for MWCNTs/PVDF-HFP membrane.

Figure 7 shows the effects of composite membranes on the flux decline behaviour during NaCl microfiltration. The permeation flow rate was measured using 2.0 g/L of an aqueous NaCl feed solution. The data indicates an initial rapid flux decline in all composite membranes, due to an increase in salt accumulation on the surface of the membranes. The flux decline is initially linked to pores blockage and later formation of a cake layer on the surface of membrane. Similar behaviour was reported elsewhere while monitoring the flux behaviour of bovine serum albumin (BSA) [35]. Interestingly, the addition of either Ag nanoparticles or MWCNTs increased the fouling resistance of the PVDF-HFP membrane, which correlate with both the pore sizes (Table 1) and contact angles of the composite.

However, the combination of both Ag nanoparticles and MWCNTs does not further promote fouling resistance of PVDF-HFP membranes, which can be linked to pore blockage by Ag nanoparticles (See Table 1) and the observed water contact angle (Figure 6). The fouling resistance rates of the composites increased as follows: PVDF-HFP < Ag-MWCNTs/PVDF-HFP < Ag/PVDF-HFP < MWCNTs/PVDF-HFP (Table 1). The fouling resistance rate of MWCNTs/PVDF-HFP composite membrane of 4.55 × 10^−2^ L·m^−2^·min^−2^ is higher than of the pore blockage/cake filtration model membrane and nanofibrous composite-PVDF-hyper branched membrane (based on the flux rate decline, the filtration model and branched membrane gave values of 2.70 × 10^−5^ and 9.23 × 10^−4^ L·m^−2^·min^−2^, respectively, as calculated from graph [36,37,38]. The MWCNTs/PVDF-HFP membrane was reused 3 times with back-wash cleaning using distilled water without significant changes in the filtrate flux (Figure 7).

#### 3.3.2. Salt Rejection Experiment

**Figure 8:**
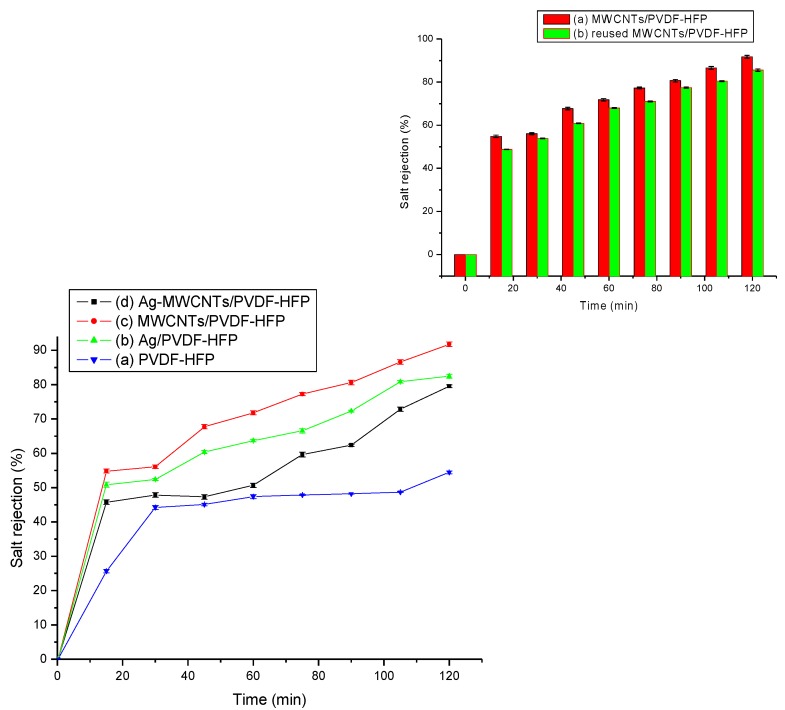
Salt rejection tests of the permeate solution after filtration using (**a**) PVDF-HFP, (**b**) Ag/PVDF-HFP, (**c**) MWCNTs/PVDF-HFP (**d**) Ag-MWCNTs/PVDF-HFP membrane and the insert showing the 1st and 3rd filtration cycles of salt rejection using MWCNTs/PVDF-HFP membrane.

Figure 8 shows the NaCl (2.0 g/L) salt rejection of the composite membranes. The salt rejection studies were undertaken by measuring conductivity of the permeate solution as time changes.

#### 3.3.3. Effects of Membrane Structure on Microbial Load Reduction

Table 2 shows the water filtration tests of composite membranes. The data indicated 100% reduction of the bacteria, on both Ag-MWCNTs/PVDF-HFP and MWCNTs/PVDF-HFP composite membranes after filtration. Ag/PVDF-HFP showed 87% reduction followed by 67% microbial reduction for PVDF-HFP membrane. It is evident from these results that the pore sizes of Ag-MWCNTs/PVDF-HFP and MWCNTs/PVDF-HFP membranes were optimum for entrapment of bacteria while allowing water to easily pass through.

#### 3.3.4. Evaluation of Antibacterial and Non-Leaching Properties of PVDF-HFP Composite Membranes

**Figure 9:**
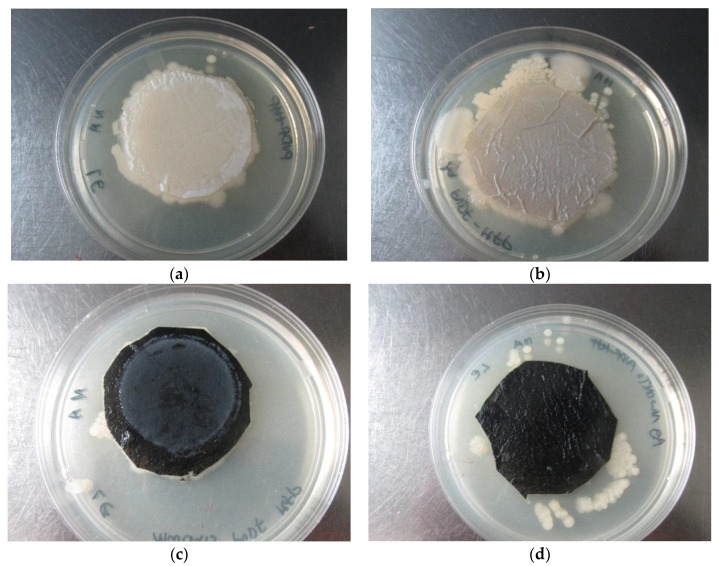
Evaluation of antibacterial activity of (**a**) PVDF-HFP, (**b**) Ag/PVDF-HFP, (**c**) MWCNTs-PVDF-HFP (**d**) Ag-MWCNTs-PVDF-HFP.

There was no bacterial growth on Ag-MWCNTs/PVDF-HFP membrane following vacuum filtration of *E. coli* spiked water (Figure 9d). This finding can be attributed to the presence of Ag nanoparticles dispersed on the surface of MWCNTs. Similar findings were reported on Ag loaded polyethersulfone (PES) hollow fibre membrane [18]. The Ag nanoparticles are not present on MWCNTs/PVDF-HFP composite; hence confluent *E. coli* was observed (Figure 9c). Furthermore, the membrane showed good non-leaching properties of the dopant materials (MWCNTs and Ag), as evidenced by bacterial growth on the edges of the membranes (Figure 9c,d).

The leaching studies of Ag were conducted on filtrates with an ICP-OES technique (after 2 h of filtration, collecting approximately 2 L of water), using silver doped membranes. Interestingly, the control tap water analysed contained 0.0228 ± 0.013 mg/L of Ag, which is below permissible levels from WHO [5]. The filtrates analysis which has passed through Ag/PVDF-HFP and Ag-MWCNTs/PVDF-HFP membranes gave 0.0275 ± 0.016 and 0.0257 ± 0.015 mg/L of Ag, respectively. This indicates that the silver content in the filtered water increased by 20% for Ag/PVDF-HFP and 13% for Ag-MWCNTs/PVDF-HFP. The results indicate that the membranes leach out very small amounts of Ag, as compared to the amount loaded on the membranes and the leached amounts are still below the acceptable limits of WHO (i.e., 0.1 mg/L Ag in drinking water) [5].

These results contradict the work reported in the literature [10,21]. This makes Ag-MWCNTs/PVDF-HFP composite membrane an ideal water purification membrane wherein dopant materials do not leach out.

**Figure 10:**
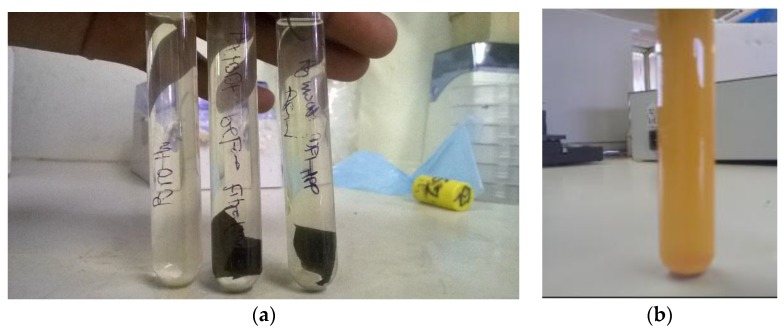
Demonstration of bactericidal property of Ag-MWCNTs/PVDF-HFP membrane in (**a**) *nutrient* broth media and (**b**) growth of *E. coli* bacteria in nutrient broth medium.

The antibacterial activity of Ag-MWCNTs/PVDF-HFP membrane was further confirmed by incubating the membrane in nutrient broth for 24 h (Figure 10). No growth was observed as indicated by non-turbid broth media in the test sample (Figure 10a), while growth was observed as cloudiness in the reference sample containing *E. coli* bacteria (Figure 10b).

## 4. Conclusions

PVDF-HFP composite membranes with either silver nanoparticles and/or MWCNTs were successfully prepared by phase inversion as confirmed by XRD and EDX data. SEM images showed that the PVDF-HFP composite membranes have high porosity and interconnected pore structures, with an average membrane diameter of approximately 180 µm. Ag and MWCNTs grafting was effective for improving membrane surface hydrophilicity, as demonstrated by swellability, water content, porosity and water contact angle changes. MWCNTs doped PVDF-HFP membrane showed up to 100% microbial load reduction, high fouling resistance rate (0.0455 ± 0.009 L·m^−2^·min^−2^) and 92% salt rejection. Filtration studies indicated that Ag-MWCNTs/PVDF-HFP membranes displayed good microbial load reduction (100%), and excellent bactericidal effects, since no growth was observed on the surface of membrane. The membrane further displayed good non-leaching properties, as evidenced by bacterial growth on the edges of the membranes and Ag leaching studies. Ag nanoparticles, MWCNTs and their combinations are suitable candidates for the improvement of PVDF-HFP membrane surface hydrophilicity and antifouling performance under real water purification conditions.

## Figures and Tables

**Table 1:** Structural features and fouling resistance rate of PVD-HFP and composite membranes.

Type of Membrane ^1^	Swellability (%)	Water Content (%)	Porosity (%)	Fouling Resistance Rate ^2^ (L·m^−2^·min^−2^)
PVDF-HFP	12	61	70	0.0233 ± 0.006
Ag/PVDF-HFP	13	67	82	0.0257 ± 0.0032
Ag-MWCNTs/PVDF-HFP	16	87	85	0.0376 ± 0.005
MWCNTs/PVDF-HFP	20	86	91	0.0455 ± 0.009

^1^ Thickness of all membranes is 180 µm, with an estimated surface area of 0.00126 m^2^. ^2^ Fouling resistance rates were calculated from the gradient of curves in Figure 7.

**Table 2:** Effects of membrane compositions on filtration of *E. coli*.

Membrane	Pre-Filtration Colony Count (CFU/100 mL)	Post-Filtration Colony Count (CFU/100 mL)	% Microbial Load Reduction
PVDF-HFP	150	50	67
Ag-MWCNTs/PVDF-HFP	150	0	100
MWCNTs/PVDF-HFP	150	0	100
Ag/PVDF-HFP	150	20	87
